# Electrical impedance tomography in perioperative medicine: careful respiratory monitoring for tailored interventions

**DOI:** 10.1186/s12871-019-0814-7

**Published:** 2019-08-07

**Authors:** Elena Spinelli, Tommaso Mauri, Alberto Fogagnolo, Gaetano Scaramuzzo, Annalisa Rundo, Domenico Grieco Luca, Giacomo Grasselli, Carlo Alberto Volta, Savino Spadaro

**Affiliations:** 1 Dipartimento di Anestesia, Rianimazione ed Emergenza-Urgenza, Fondazione IRCCS Ca’ Granda Ospedale Maggiore Policlinico, Università degli studi di Milano, Milan, Italy; 2 Department Morphology, Surgery and Experimental medicine, Anesthesia and Intensive care section, University of Ferrara, Azienda Ospedaliera- Universitaria Sant’Anna, 8, Aldo Moro, Ferrara, Italy; 3 UOC Anestesia e Rianimazione, Polo ospedaliero Belcolle ASL, Viterbo, Italy; 4 Department of Anesthesiology and Intensive Care Medicine, Catholic University of the Sacred Heart, Fondazione “Policlinico Universitario A. Gemelli”, Rome, Italy

**Keywords:** Electrical impedance tomography, Perioperative medicine, Hemodynamic monitoring, Non-operating room anaesthesia

## Abstract

**Background:**

Electrical impedance tomography (EIT) is a non-invasive radiation-free monitoring technique that provides images based on tissue electrical conductivity of the chest. Several investigations applied EIT in the context of perioperative medicine, which is not confined to the intraoperative period but begins with the preoperative assessment and extends to postoperative follow-up.

**Main body:**

EIT could provide careful respiratory monitoring in the preoperative assessment to improve preparation for surgery, during anaesthesia to guide optimal ventilation strategies and to monitor the hemodynamic status and in the postoperative period for early detection of respiratory complications. Moreover, EIT could further enhance care of patients undergoing perioperative diagnostic procedures. This narrative review summarizes the latest evidence on the application of this technique to the surgical patient, focusing also on possible future perspectives.

**Conclusions:**

EIT is a promising technique for the perioperative assessment of surgical patients, providing tailored adaptive respiratory and haemodynamic monitoring. Further studies are needed to address the current technological limitations, confirm the findings and evaluate which patients can benefit more from this technology.

## Background

Electrical impedance tomography (EIT) is a non-invasive radiation-free monitoring technique that provides images based on changes in the electrical conductivity of body sections. During the latest years, EIT has been applied to several fields. Within the chest, impedance changes dynamically due to changes in lung volume (e.g., during respiration) and/or due to changes in blood and fluids content (e.g., within the heart during cardiac cycle). Trough digital reconstruction, EIT transforms the electrical signal in a dynamic image that can be shown at the bedside [1]. Thus, lung function and central haemodynamics monitoring are ideal and promising fields of application for EIT.

The care of surgical patients is not limited to the time of surgery but it’s extended to the preoperative assessment and postoperative monitoring. Preoperative evaluation allows to identify factors that may need to be corrected to improve patients’ outcome. Intra-operative monitoring allows to personalize interventions based on the patients’ characteristics with the aim to reduce postoperative complications. Postoperative monitoring extends outside of the operating theatre to the Intensive Care Unit and to ambulatory procedures.

In this narrative review, we will summarize the current evidences on the application of EIT in the perioperative care of surgical patients (Table 1). Different EIT models are commercially available (Table 2). EIT has been successfully used a) to improve the quality of preoperative lung functional tests, b) to evaluate the changes in lung volumes during anaesthesia, c) to obtain non-invasive haemodynamic monitoring and d) to increase the early recognition of postoperative complications. Finally, application of this technology to support perioperative procedures will be analysed.

**Table 1:** EIT derived monitoring tools and corresponding clinical implications. In the table are synthetized the EIT derived tools and the corresponding clinical implication in the different settings (preoperative, intraoperative, postoperative, non-operative room anesthesia) of surgical patient’s care

Monitoring tools	Clinical implication
Pre-operative	
Test bronchodilator reversibility in asthma patients (Frerichs, 2016)	Help pharmacological management
Identifying patients at higher risk of expiratory airflow limitation (Vogt, 2016)	Risk stratification, MV management
FRC reduction during induction (Humphreys, 2011)	Choice of pre-oxygenation strategies
Intraoperative use for ventilatory setting	
Mechanical proprieties of non-dependent and dependent lung regions (Pereira, 2018)	Choice of Positive end-expiratory pressure Prevention of postoperative atelectasis
Silent spaces (Spadaro, 2018)	Choice of Positive end-expiratory pressure
Regional ventilation delay index (Nestler, 2017)	Choice of Positive end-expiratory pressure Response to recruitment maneuvers
End Expiratory Lung Impedance (Erlandsson, 2006)	FRC estimation Choice of Positive end-expiratory pressure
Left and right lung ventilation distribution (Steinmann, 2008)	Confirm the correct position of a double lumen tube
Intraoperative use for hemodynamic monitoring	
Dynamic changing in stroke volume (Vonk-Noordegraaf, 2000)	Fluids responsiveness assessment Pharmacological management
Cyclic impedance changes in the descending aorta (Maish, 2011)	Stroke volume variation assessment
Peri-extubation and postoperative	
Postoperative atelectasis (Pereira, 2018)	Indication for postoperative non-invasive ventilation Identifying the need for early mobilization
Distribution of ventilation after endotracheal suctioning (Heinze, 2011)	Efficacy of endotracheal suctioning
Changes in regional expiratory time constant (τ) (de La Oliva, 2017)	Early identification of bronchospasm Pharmacological management
Non-operative room anesthesia	
Real-time assessment of pulmonary vasodilators effects (Frerichs, 2016)	Pharmacological management of patients with chronic pulmonary hypertension
Identifying the site of “blind” broncho-alveolar lavage (Grieco, 2016)	Detect with precision the site of lung sampling
Out-of-phase changes in the impedance during tidal breathing (Bläser, 2014)	Pleural effusion assessment

**Table 2:** Summary of EIT devices used in the papers discussed in the review

Study	Device	Commercially available (Y/N)	Number of electrodes	Image reconstruction type
Frierichs, 2016	Goe-MF II EIT device (CareFusion, Höchberg, Germany)	N	16	GREIT; Back-projection algorithm (Krause, 2014)
Vogt, 2012				
Vogt, 2016				
Krause, 2014				
Frerichs, 2019				
Bläser, 2014				
Erlandsson, 2006	Dräger/GoeMFII, Lübeck, Germany	N	–	–
Nestler, 2017	PulmoVista 500; Dräger Medical, Lübeck, Germany	Y	16	–
Eronia, 2017				
Zhao, 2018				
Grieco, 2016				
Becher, 2018				
Schaefer, 2014	EIT Evaluation Kit 2, Dräger Medical, Lübeck, Germany	N	32	–
Steinmann, 2008				
Zhao, 2009				
Zhao, 2013				
Pereira, 2018	Enlight 1800, (Timpel, Brazil)	Y	32	–
da Silva Ramos, 2018				
Spadaro 2018	Swisstom BB^2^, Swisstom AG, Landquart, Switzerland	Y	32	–
Karagiannidis, 2018				
Braun, 2018				
Reinius 2015	Enlight impedance tomography monitor (Timpel, Brasil)	N	32	
Yoshida, 2013	Enlight impedance tomography monitor (Dixtal, Brasil)	N	32	3-D finite element mesh and a linearized sensitivity-matrix algorithm
Alves, 2014				
Vonk-Noordegraaf, 2000 Smit, 2002	Sheffield Applied Potential Tomograph (DAS-01P portable data acquisition system, mark I, IBEES, Sheffield, UK)	N	16	–
Maisch, 2011	Enlight (Dixtal, Sao Paulo, Brazil)	N	32	–
Karsten, 2014	EIT (Dräger Medical AG, Germany)	N	32	–
Rossi F, 2013	Enlight® technology model DX-1800 (Dixtal, Sao Paulo, Brazil)	N	16	3-D finite element mesh
De La Oliva, 2017	Customized textile belt and a computed tomography-based patient-specific 3-dimensional chest model (Swisstom, Landquart, Switzerland	N	–	–
Frerichs 2019	Goe-MF II EIT system; Viasys Healthcare, Höchberg, Germany	N	–	–

In the table are reported the devices, number of electrodes, commercial availability and reconstruction algorithm of the EIT devices used in the papers discussed in the current review

## Methods

An extensive research was carried out from January 2018 to March 2019 using the main databases (Pubmed, Embase, Scopus) to identify papers dealing with the use of EIT in the preoperative, intraoperative and postoperative period. Only English papers published on peer review indexed journals were taken into consideration. The results of this search are reported in this narrative review with reference to the author’s clinical and scientific experience.

## Results

### EIT imaging principles

Electrical impedance tomography is a non-invasive, radiation-free imaging technique based on the different capability of body tissues to conduct electricity. Specifically, the explored area of the body is surrounded by electrodes (generally from 16 to 32), recording the surface voltage after the repeated injection of small amount of currents (generally 5 mA) at a frequency of 70–80 kHz [2]. The electrodes are usually placed on belts and each pair of consecutive electrodes is alternatively used to inject current or measure the surface voltage. The different voltages recorded at the surface are therefore reconstructed in a binary map (image) using different and dedicated algorithms [3]. This concept can be applied in static (absolute EIT, a-EIT) [4] or dynamic situations (functional EIT, f-EIT) [5] With functional EIT, that is the most commonly used technique in the clinical practice, the change of impedance during time (∆Z) is reconstructed in a color-code dynamic image, usually available today at the patients’ bedside. Several body parts have been explored using EIT, e.g. lungs and heart (chest EIT), breast [6, 7] and brain [8], being lungs - and more recently heart - the most explored ones. The impedance maps depend on the algorithm used to convert the raw current data into images. Several algorithms have been developed during the last years [3] to optimize image reconstruction and to face the so called “inverse problem”, i.e. the process of calculating the internal conductivity of a body by measuring the surface voltage [9, 10]. Actually, the final user has no possibility to change the reconstruction algorithm which depends on the manufacturer, but recent evidences suggest that the different algorithms can result in similar EIT derived parameters [11].

### Role of EIT in the preoperative assessment

Pre-operative EIT imaging has the potential to give relevant information to anesthesiologists: this can influence their strategy during the peri-operative time. EIT imaging is becoming an integrative tool to conventional pulmonary preoperative tests in patients affected by chronic obstructive pulmonary disease (COPD) and asthma [1]. Specifically, EIT showed a high and fast sensitivity to bronchodilator reversibility test, thus potentially influencing perioperative pharmacological management both in asthma [12] and COPD [13] patients by recognition of less severely obstructed patients. Since EIT can determine the spatial and temporal heterogeneity of ventilation in COPD patients, [14] it could influence perioperative mechanical ventilation (MV) settings by identifying patients at higher risk of expiratory airflow limitation (EFL) and pulmonary hyperinflation, who might require longer expiratory time and more aggressive aerosol therapy. Indeed, both EFL and hyperinflation have been showed to increase the risk of postoperative pulmonary complications [15] and therefore the early recognition of patients at higher risk could potentially reduce perioperative morbidity.

### Role of EIT during anesthesia induction

Functional residual capacity (FRC) is defined as the volume of gas remaining in the lungs at the end of expiration, in resting conditions (zero positive end-expiratory pressure). While it is well-known that FRC is decreased during induction of anesthesia [16], the degree and the variability of this reduction is less studied, probably due to its difficult estimation [17]. One of the aims of mechanical ventilation should be to keep the FRC values as similar as possible to preoperative values; indeed, a reduction in FRC below the closing volume will result in altered blood gases by increased of ventilation/perfusion mismatch [18–20]. Conversely, raising the FRC above the physiological values can increase the lung volume to a level close to total lung capacity during, potentially increasing the stress imposed on the lung [21]. Currently, stress and strain cannot be easily evaluated at the bedside because they cannot be inferenced based on ventilatory targets derived from respiratory mechanics, such as tidal volume per kilogram of body weight or plateau pressure [22]; furthermore, the same limitations applies to the driving pressure, a parameter recently advocated as a target for ventilator settings, which however reflects dynamic strain only when a very low (≤2 cmH_2_O) positive end-expiratory pressure (PEEP) is used [17]. These considerations prompt a search for bedside-available tools to assess anesthesia-induced aeration loss and intraoperative functional residual capacity, which could guide individualized ventilator settings. According to the recent literature, EIT imaging might play a role in this context.

**Fig. 1:**
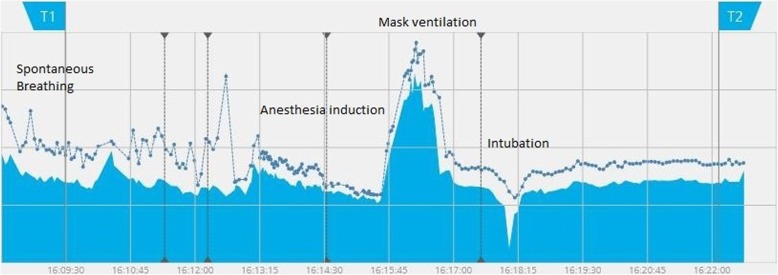
End expiratory lung impedance change during anesthesia induction. Evaluation of End-Expiratory Lung Impedance (EELI, continuous blue line) and End Inspiratory Lung Impedance (EILI, blue dots, non-continuous blue line) during different moments of general anesthesia induction in a 65 years old patient undergoing laparoscopic cholecystectomy. Note decrease of TV and EELV during anesthesia induction and increase in EELV during mask ventilation with PEEP. Finally, stable ventilation and EELV were reached after intubation and start of control mechanical ventilation

In children undergoing cardiac surgery [23], EIT showed a decrease in FRC after anesthesia induction, which can be reversed by the application of PEEP. Similarly, EIT showed the same phenomenon in morbidly obese patients undergoing laparoscopic gastric bypass surgery [24]; in these patients, pre-oxygenation with a tight-fitting mask and 10 cmH_2_O-PEEP was able to ensure a transient increase in FRC, useful to avoid hypoxia during anesthesia induction (Fig. 1). On the other hand, Nestler et al. demonstrated how bag-mask ventilation without PEEP results in significant decrease in post-intubation FRC [25]. Therefore, EIT seems a promising tool to monitor the efficacy of different pre-oxygenation strategies. Of note, an ongoing trial (NCT03615417) is using EIT to evaluate if High Flow Nasal Cannula (HFNC) can prevent FRC impairment during anesthesia induction.

### Role of intraoperative EIT imaging to guide mechanical ventilation

**Fig. 2:**
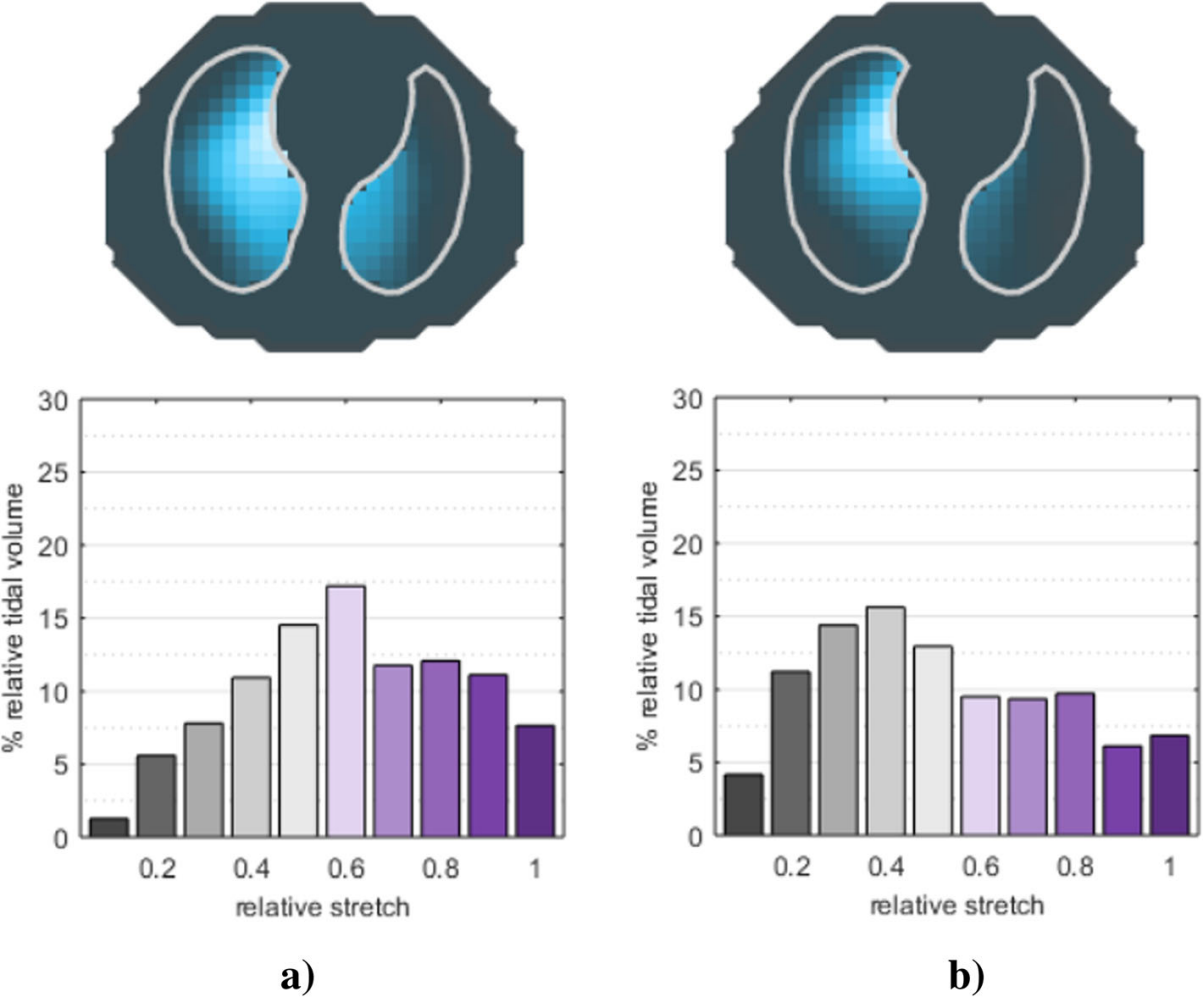
Tidal volume distribution during spontaneous breathing (**a**) and mechanical ventilation (**b**). Tidal image (top) and relative stretch distribution (bottom) during spontaneous breathing (**a**) and after intubation and start of controlled mechanical ventilation (**b**) in the same patient undergoing general anesthesia. During mechanical ventilation, tidal volume is redistributed toward the ventral lung; moreover, hypoventilated areas increase, as shown by the relative stretch histogram (lower values indicate smaller regional tidal volume)

Electrical impedance tomography can evaluate lung function during general anesthesia by imaging breath by breath changes in ventilation distribution (Fig. 2), helping physicians in tailoring mechanical ventilation [26]. In the last few years, different approaches have been tested to individualize intraoperative mechanical ventilation according to EIT, particularly focusing on PEEP titration.

Recently, a method consisting in finding the best mechanical compromise between non-dependent and dependent lung regions has been proven to enhance intraoperative oxygenation and limit driving pressure, minimizing both overdistension and collapse [27]. Interestingly, Pereira et al. showed that this EIT-guided PEEP setting protocol can also benefit postoperative respiratory function, by preventing development of postoperative atelectasis, as assessed by computed tomography. These beneficial effects are more pronounced in patients undergoing laparoscopy in comparison to laparotomic procedures.

Nestler et al. [25] used another EIT-derived parameter to set PEEP in obese patients undergoing general anesthesia: the regional ventilation delay index (RVDI), calculated as the standard deviation of all pixels’ regional ventilation delay, which reflects the occurrence of cyclic tidal-induced recruitment and collapse. After a recruitment maneuver, PEEP titrated to minimize RVDI yielded significant improvement in gas exchange and regional homogeneity when compared to fixed amount of PEEP (5 cmH_2_O) but didn’t impact post-operative parameters.

The effects of PEEP variation on lung recruitment or overinflation can be evaluated monitoring End Expiratory Lung Impedance (EELI). The latter represents the value of impedance at the end of expiration and the change in EELI should reflect the lung-volume change due to PEEP. Erlandsson et al. [24] demonstrated that, in obese patients undergoing laparoscopic surgery, an increase/decrease of EELI slope following a change of PEEP, indicates respectively recruitment/derecruitment; while a horizontal tracing corresponds to a stable end-expiratory lung volume, i.e. the optimal PEEP. Mauri et al. [28], albeit in acute respiratory failure patients, showed that it is possible to measure recruitment by subtracting from the measured EELI change between two PEEP levels the amount of impedance increase expected from the compliance at lower PEEP. Conversely, in patients undergoing general anesthesia, lung overinflation could be suspected if, after increasing PEEP, the increase in EELI is less than the one predicted by the compliance lower PEEP. An important issue still needed to be addressed in using EELI intraoperatively is the relative high number of electrical interferences determined by the monitoring system.

EIT has been used to confirm the correct positioning of double lumen tubes (DLT) and to adjust tidal volume/PEEP combinations in patients requiring one lung ventilation (OLV). Furthermore, although the gold standard for routine check of the correct positioning of the DLT is fiberoptic bronchoscopy, EIT can non-invasively recognize the initial misplacement of DLTs in the contralateral main bronchus by accurately displaying left and right lung ventilation [29]. When shifting from two lung ventilation (TLV) to OLV, the mechanical properties of the ventilated lung change. Lateral position and the exclusion of one lung from ventilation determine a change in lung compliance, resistances and in tidal volume distribution. Hence both tidal volume (TV) and PEEP should be re-adjusted during the surgery, adding complexity to the ventilatory management of these patients [19]. All these changes can be detected by EIT: by using an index of inhomogeneity derived from EIT (GI, global inhomogeneity index), Zhao et al. [30] showed that it is possible to titrate the combination of PEEP and TV in patients shifting from TLV to OLV. In their study, the GI correlated with the gas distribution in the lung, revealing good inter-patient comparability. The same authors recently explored if regional ventilation distribution (measured by EIT) and PaO_2_ could help titrating TV and PEEP during OLV [31].

Setting the right PEEP can be relevant for intraoperative and postoperative patient’s care. During general anesthesia, more than 90% of patients develop atelectasis, primarily due to a reduction in FRC [16, 18]. As previously pointed out in the present review, the amount of intraoperative FRC loss is unpredictable and inconstant, being not only influenced by patient’s characteristics (BMI, lung function, age) or anesthesia induction technique, but also by intraoperative variables such as positioning (e.g. Trendelenburg position) and surgical procedures (i.e. capnoperitoneum). Since we were able to assess the real-time FRC loss by using EIT, as shown in a representative patient (Fig. 1), this monitoring could potentially allow to set a dynamic individualized PEEP level, adapting to the changes throughout the entire surgical procedure.

Hypo-ventilated areas suggestive of atelectasis – the so called “Silent Spaces” [32] – have been identified by Ukere et al. using EIT in awake and anesthetized patients in different body positions. Since atelectasis can deteriorate gas exchange - by rising shunt fraction - and increase the risk of postoperative complication - being a possible focus of infection – one might expect that silent spaces monitoring could improve intraoperative patient’s care if PEEP is set to reduce their amount.

EIT could be helpful during anesthesia not only to minimize atelectasis, but also because it is able to measure regional flow (i.e the airflow that inflates the different part of the lung, according to their time constant). This technique could be useful to explore poorly understood phenomenon like regional expiratory flow limitation [33]. This could be of clinical relevance since EFL is associated with poor outcomes such as increased risk of postoperative pulmonary complications, extubation failure, and airway injury in acute respiratory distress syndrome [34, 35]. How EFL is distributed regionally during general anesthesia is far from being understood and EIT could add interesting insights into its comprehension.

Another interesting aspect that should be evaluated during general anesthesia is the ratio between ventilation and perfusion. The EIT guided lung perfusion monitoring, using hypertonic saline injection, will open new possibilities to titrate MV targeted on ventilation/perfusion mismatch. However, till now, the feasibility of intraoperative evaluation of ventilation and perfusion distribution has been explored only in experimental models of OLV with promising results [36].

Despite the usefulness of EIT in evaluating intraoperative ventilation has been highlighted by an increasing number of trials, still no RCTs has shown a direct correlation between EIT use and postoperative complications, especially the pulmonary postoperative ones (PPC). This can be explained by the adequate sample size required to address this outcome [37] and the still high costs and professional support needed to enroll a high number of patients in studies using EIT.

### Intraoperative use of EIT for hemodynamic monitoring

Improving monitoring accuracy while minimizing invasiveness are two conflicting goals of intra-operative hemodynamic management in the era of the “enhanced recovery after surgery” approach [38]. Goal-directed therapy improves surgical outcomes and decreases the risk of post-operative complications [39, 40]; therefore, intra-operative fluid administration and use of vasoactive drug should be guided by the dynamic assessment of cardiac function and the detection of fluid responsiveness. On the other hand, routine use of advanced monitoring modalities in surgical patients is hindered by the risks of complications [41].

EIT could provide a non-invasive alternative for hemodynamic monitoring during surgery. Vonk-Noordegraaf showed that changes of heart blood volume assessed by EIT could be used to measure stroke volume (SV) in clinically stable patients [42]. This was confirmed in an experimental study, in which changes in cardiac impedance measured by EIT correlated with SV assessed by transpulmonary thermodilution [43]. It must be noticed that an acceptable accuracy in the estimation of SV by EIT could be achieved in both studies only when calibration was performed against a reference method (i.e. thermodilution). This could be a major limit to the use of EIT for non-invasive hemodynamic monitoring. Nevertheless, obtaining the absolute value of SV might not be essential for intra-operative monitoring. Indeed, assessing the relative changes of SV induced by a challenge (for example a fluid bolus) might be enough to guide hemodynamic management. Accordingly, in an experimental model of hemorrhagic shock and volume resuscitation, in which changes in systolic impedance within the lung were used to track changes in SV, EIT demonstrated acceptable trending monitoring [44].

The possibility to assess dynamic indexes of fluid responsiveness by EIT has been tested in experimental studies. Stroke volume variation (SVV) is a dynamic parameter that predicts fluid responsiveness: the cyclic changes in SV induced by ventilation (heart-lung interaction) are greater when the heart is working on the steep part of the Starling curve; therefore, values of SVV above a threshold indicate that an increase in preload will result in an increase in stroke volume (fluid responsiveness) [45]. Currently, dynamic indexes of fluid responsiveness cannot be reliably assessed with non-invasive techniques under conditions of hemodynamic instability [46, 47]. A pioneering experimental study demonstrated the ability of EIT to assess left ventricular SVV by cyclic impedance changes in the descending aorta, over a wide range of volemic conditions and ventilation settings [48].

The application of EIT for hemodynamic monitoring is a novel field of research with promising, but still preliminary, results. The use of EIT in evaluating SV assumes that the EIT heart signal depends from the ventricular blood flow. However,, the identification of the heart related EIT signal is not well defined, being for example difficult to separate, on the EIT transverse plain, the ventricles from the atria [42] or from other structures anatomically close to the heart e.g. the aorta or the pulmonary arteries [49] Moreover, changes in body and heart position [50], lung volume and hematocrit are expected during surgery and could affect EIT signal [51]. The reliability of not calibrated measurements has to be investigated during hemodynamic instability. Interference with the electromagnetic operating system and pneumoperitoneum could prevent continuous intra-operative monitoring. Despite these potential limitations, EIT has the advantages of being non-invasive, providing dynamic real-time measurements, lacking operator-dependency [52] and allowing continuous monitoring also during the postoperative time.

### Role of EIT monitoring during the post-operative period

While atelectasis can be enhanced after anesthesia induction and during surgery due to body position, fluid therapy or pneumoperitoneum, it should be considered that the risk of atelectasis is still high immediately after the end of surgery and / or after extubation. The main reasons are the persistency of anesthesia, with reduced inspiratory effort and transpulmonary pressure, impaired cough reflex and reduced ability to clear secretions due to residual paralysis and/or poor pain control. This could be responsible of postoperative pulmonary complications. Thus, changes in ventilation distribution and decreased EELI measured by EIT might be early and sensitive signs of post-operative atelectasis.

Schaefer et al. [26] described the feasibility to monitor regional tidal volume distribution before the induction of anesthesia, intra-operatively, after extubation and in the post-operative care. The authors showed that tidal ventilation shifts towards the ventral part of the lungs during general anesthesia with paralysis. When spontaneous breathing is restored and after extubation, ventilation and re-aeration of the dorsal part of the lungs were restored, with homogenous ventilation indicating healthy lungs and no atelectasis. Even using personalized intra-operative PEEP setting and a recruitment maneuver before extubation, early post-operative EELV is lower than before induction of anesthesia [25]. Since a decrease in EELV might increase the risk of developing postoperative atelectasis, EIT monitoring could support identifying obese patients with reduced EELV who might benefit from non-invasive ventilation and early mobilization. Indeed, Reychler et al. [53] described the positive effects of early physiotherapy including incentivized spirometry and the application of PEEP and tidal volume in early post-surgical patients. Karsten et al. used EIT to evaluate the impact of low versus high PEEP during laparoscopic surgery on post-operative ventilation distribution and showed that higher intra-operative PEEP induced more homogeneous ventilation distribution in the early post-operative period [54].

Few studies [55, 56] assessed the feasibility, accuracy and effectiveness of monitoring the respiratory pattern of postoperative patients by simplified electrical impedance devices. This technology does not allow monitoring of regional distribution of ventilation as electrodes are positioned only in the anterior part of the chest, but it gives accurate non-invasive continue estimates of the respiratory rate and the tidal volume. In this way, a reduction of minute ventilation can be detected before they cause peripheral oxygen desaturation or increase in EtCO_2_.

EIT also might be helpful to check the effects of endotracheal suctioning, a maneuver that is widely applied before extubation. Heinze et al. [57] described ventral shift in distribution of ventilation after suctioning, likely indicating dorsal de-recruitment. Interestingly, the shift in ventilation was correlated with decreased EELV after suctioning. In patients showing such changes, a recruitment might be indicated after suctioning before extubation.

Similar potentiality for EIT use in the early post-operative period were reported in the pediatric field. Krause et al. [58] described shift of tidal volume distribution towards dorsal lung regions after extubation in a population of infant and children undergoing cardiac surgery, thus confirming data reported in adults. In preterm infants, Rossi et al. [59] further showed how ventilation homogeneity measured by EIT might guide prophylactic application of CPAP after extubation.

Bronchospasm is another complication of the early post-extubation period, especially in patients with history of asthma, COPD or hyper-reactive airways such as pediatric patients. De la Oliva et al. [60] used EIT to monitor changes in global and regional expiratory time constant (τ) in a pediatric patient early after liver transplantation. The authors showed by EIT inhomogeneous distribution of increased τ during bronchospasm with shift of ventilation and air trapping in the ventral lung and rapid decrease of τ and inhomogeneity after suctioning and salbutamol injection.

### EIT monitoring during perioperative diagnostic procedures

Because of its simplicity to use, radiation-free and capability to provide dynamic measurements, EIT has been proposed as a monitoring tool in a variety of clinical scenarios outside the operating room.

As already mentioned, chest EIT can aid ambulatory pulmonary function testing in patients with chronic respiratory diseases; integrating results from conventional flow/volume derived indices and chest imaging allows to identify spatial and temporal heterogeneities in regional ventilation [13, 61], with the possibility of real-time assessment of pharmacological induced changes [12]. In this sense, EIT has also been proven to reliably detect in real time the vascular effects of pulmonary vasodilators in patients with chronic pulmonary hypertension [62].

**Fig. 3:**
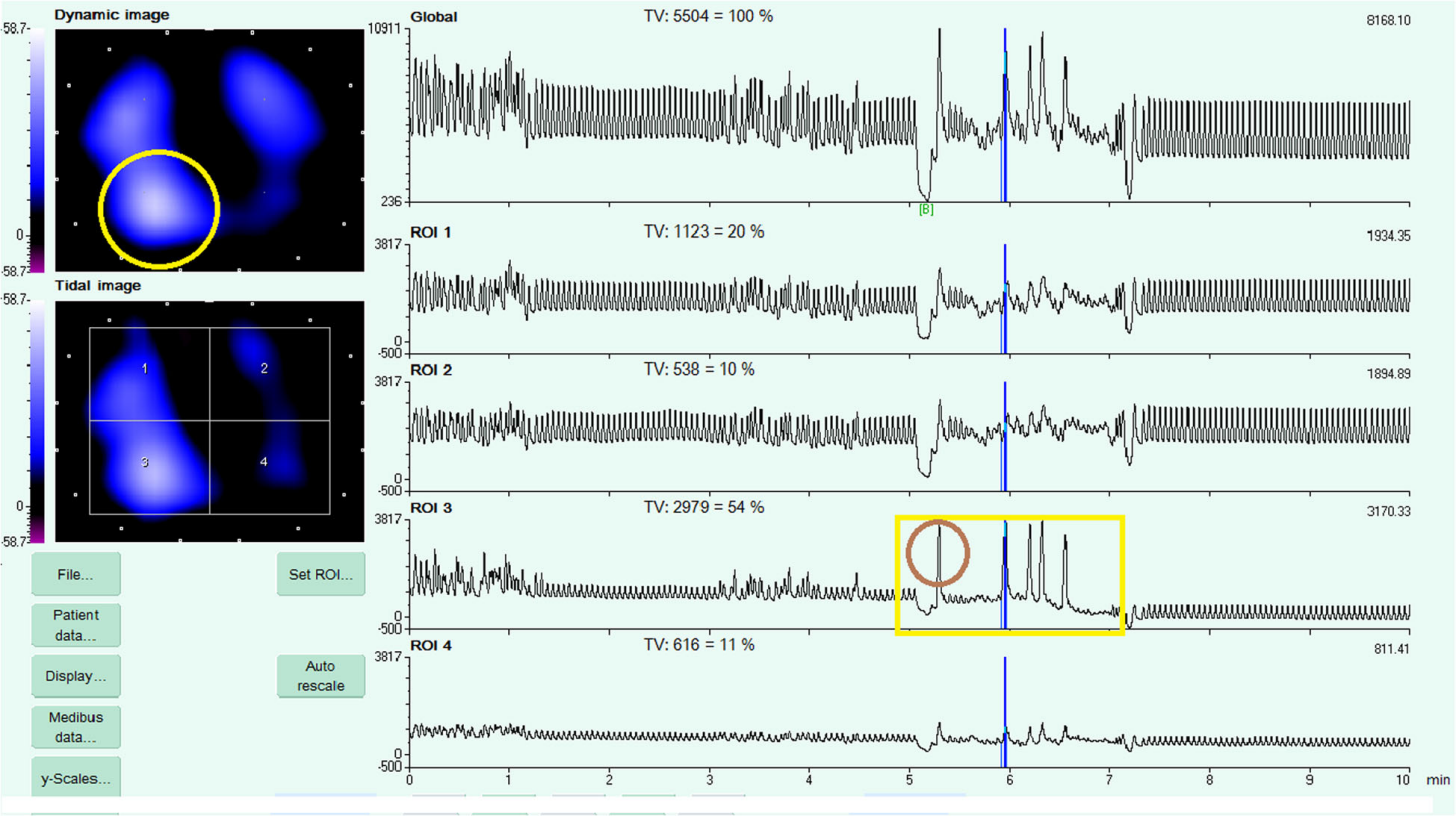
Electrical impedance tomography (EIT) image acquired during a non-bronchoscopic bronchoalveolar lavage (blindBAL) performed in the dorsal lung. (Grieco et al., Intensive Care Med (2016) 42: 1088)

EIT has been proposed to monitor the site of sampling during non-bronchoscopic ‘blind’ broncho-alveolar lavage (BAL) for pneumonia diagnosis (Fig. 3) [63]. Non-bronchoscopic BAL is a simple, manageable and cost-effective alternative to BAL with flexible bronchoscopy; however, some authors suggest that it is wise to perform microbiological lung sampling in an area where radiological infiltrates are known to be present, warranting tools to precisely identify the exact site of the lavage in case of a non-bronchoscopic procedure [64]. In a recent study conducted on piglets, EIT signals from more than one hundred BAL procedures were systematically analyzed: EIT efficiently identifies the regions where saline is inflated by means of an abrupt a decrease in chest impedance [65]. This study was conducted during apnea and saline insufflation yielded decrease in lung impedance: a previous report showed that, if the procedure is performed in humans receiving mechanical ventilation, saline insufflation rather generates increases in lung impedance in the involved region, possibly because of local redistribution of tidal volume and the coexistence of gas and liquid in the spectrum of EIT [64–66]. Both studies seem consistent in showing that EIT is capable to detect with precision the site of lung sampling during blind procedures.

In patients with pleural effusions, out-of-phase ‘paradoxical’ EIT changes (increases during expiration and decreases during inspiration) may occur during tidal breathing, likely due to an overshoot phenomenon produced by the algorithms for image reconstruction in case of proximity of areas with high and low ventilation-related impedance changes [67, 68]. Consistently, out-of-phase changes in the impedance during tidal breathing, that involve entire inspiration and hence differ from the pendelluft phenomenon (i.e. paradoxical movement of air from non-dependent to dependent lung regions in the very early phase of inspiration) [69], have been shown to be very suggestive of the presence of pleural effusions and may support clinical diagnosis [66]. Several categories of patients undergo drainage of pleural effusion outside the setting of the operating room. EIT represents a simple, bedside available tool to monitor lung re-aeration due to pleural effusion drainage. In a prospective study on 22 patients undergoing the procedure due to malignant pleural effusion, EIT had high reliability in documenting reaeration of the ipsi- and contro-lateral lung, which occurred immediately without further changes over the next hour [70].

## Limitation and future topics

Despite EIT is now available at the bedside and has been applied to several clinical scenarios, it is still a “promising” technique. In addition to the aspects already addressed in the current review, EIT must deal with some technical limitations that need to be faced in the future. Compared to other imaging techniques used in the anesthesia field like ultrasound or CT scans, EIT has a relatively low spatial resolution [71]. Moreover, the position of the electrodes can modify the obtained image, rising problems of intra and inter patient reproducibility [72]. Furthermore, EIT technology currently available at the bedside explores a 3-D transverse plain providing a 2-D image. New reconstruction techniques are being developed to render the explored tissue slice in a 3-dimensional image [73]. Finally, patients with pacemaker or implantable cardioverter-defibrillator cannot be monitored using EIT since there is no evidence about the interference between active implants and impedance tomography measurements.

## Conclusions

Electrical Impedance Tomography is a promising tool for individualizing the perioperative care of surgical patients. Its use can improve preoperative assessment, intraoperative monitoring and early recognition of postoperative complications. Besides the inherent technical limitations of this technology, several steps are required to make EIT more suitable for perioperative medicine. To facilitate the use of EIT in the operating room, development of algorithms that translate the image information into physiologic indexes will be key. The availability and portability of the machine should be increased. Moreover, if the demonstrated ability to track perioperative changes in cardiorespiratory physiology has to impact clinical outcomes, studies are needed to univocally identify parameters to be monitored, and to define reference values and therapeutic targets for each application. Anesthesiologists will need to gain experience in the acquisition and interpretation of EIT data. Further studies are therefore needed to clarify the full possibilities of this new technology before a widespread use could be safely implemented.

## Data Availability

All data supporting this manuscript are available in the text.

## Abbreviations

BAL: Bronco-alveolar lavage
BMI: Body mass index
COPD: Chronic obstructive pulmonary disease
CPAP: Continuous positive airway pressure
DLT: Double lumen tube
EELI: End expiratory lung impedance
EFL: Expiratory flow limitation
EIT: Electrical impedance tomography
EtCO_2_: End tidal CO_2_
FRC: Functional residual capacity
GI: Global inhomogeneity index
HFNC: High flow nasal cannula
MV: Mechanical ventilation
NORA: Non-operative room anaesthesia
OLV: One lung ventilation
PaO_2_: Partial pressure of oxygen in arterial blood
PEEP: Positive end-expiratory pressure
PPC: Pulmonary postoperative complications
RVDI: Regional ventilation delay index
SV: Stroke volume
SVV: Stroke volume variation
TLV: Two lung ventilation
TV: Tidal volume
τ: time constant
