# Correction to: Electrical impedance tomography in perioperative medicine: careful respiratory monitoring for tailored interventions

**DOI:** 10.1186/s12871-019-0840-5

**Published:** 2019-09-04

**Authors:** Elena Spinelli, Tommaso Mauri, Alberto Fogagnolo, Gaetano Scaramuzzo, Annalisa Rundo, Domenico Luca Grieco, Giacomo Grasselli, Carlo Alberto Volta, Savino Spadaro

**Affiliations:** 1 Dipartimento di Anestesia, Rianimazione ed Emergenza-Urgenza, Fondazione IRCCS Ca’ Granda Ospedale Maggiore Policlinico, Università degli studi di Milano, Milan, Italy; 2 Department Morphology, Surgery and Experimental medicine, Anesthesia and Intensive care section, University of Ferrara, Azienda Ospedaliera- Universitaria Sant’Anna, 8, Aldo Moro, Ferrara, Italy; 3 UOC Anestesia e Rianimazione, Polo ospedaliero Belcolle ASL, Viterbo, Italy; 4 Department of Anesthesiology and Intensive Care Medicine, Catholic University of the Sacred Heart, Fondazione “Policlinico Universitario A. Gemelli”, Rome, Italy

**Correction to: BMC Anesthesiol**

**https://doi.org/10.1186/s12871-019-0814-7**

Following publication of the original article [1], the authors reported that one of the co-authors has a mistake in the author name; the middle name and surname are switched. This is the correct information:

Surname: Grieco

First name: Domenico Luca

The original article [1] has been updated
